# Assessment of Anti-oxidative, Anti-inflammatory, and Anti-cancer Activity of Magnesium Oxide Doped Chitosan/Polyvinyl Alcohol With Catharanthus roseus: An In Vitro Study

**DOI:** 10.7759/cureus.70103

**Published:** 2024-09-24

**Authors:** Challa Hemanth, Sugumar Vimal

**Affiliations:** 1 Biochemistry, Saveetha Medical College and Hospitals, Saveetha Institute of Medical and Technical Sciences (SIMATS) Saveetha University, Chennai, IND; 2 Biochemistry, Saveetha Medical College and Hospital, Saveetha Institute of Medical and Technical Sciences (SIMATS) Saveetha University, Chennai, IND

**Keywords:** anti-cancer properties, anti-inflammatory activity, antioxidant activity, biocomposite materials, catharanthus roseus, chitosan, h2o2 test, magnesium oxide (mgo), polyvinyl alcohol (pva), scanning electron microscopy (sem)

## Abstract

Background

Recent biomedical research has emphasized the potential of biocomposite materials for medicinal purposes. This work investigates the combination of magnesium oxide (MgO)-doped chitosan and polyvinyl alcohol (PVA) with extracts from* Catharanthus roseus*, a medicinal plant renowned for its abundant alkaloid content and therapeutic advantages. The antioxidant, anti-inflammatory, and anti-cancer characteristics of this unique biocomposite material are being studied better to understand its prospective uses in biomedicine.

Aim

The goal of this study is to investigate the in vitro oxidative, anti-inflammatory, and anti-cancer properties of a biocomposite made of MgO-doped chitosan and PVA, combined with an extract from *C. roseus*.

Materials and methods

The biocomposite was made by blending chitosan and PVA in equal proportions and adding MgO nanoparticles to *C. roseus *extract. The surface morphology was analysed using scanning electron microscopy (SEM). The antioxidant activity was measured using the H_2_O_2_ test, the anti-inflammatory activity was identified using the egg albumin assay, and the anti-cancer activity was analyzed using the MTT assay on MCF-7 breast cancer cell lines. In addition, cell morphology investigations were performed to evaluate any alterations after treatment.

Results

The SEM investigation showed clearly defined and sleek nanoparticles. The biocomposite demonstrated notable antioxidant activity, with inhibition percentages escalating in proportion to the concentration. The anti-inflammatory assays demonstrated inhibition percentages comparable to diclofenac, reaching approximately 90% at the maximum concentration. The MTT experiment revealed that the viability of MCF-7 cells decreased in a manner that was dependent on the dose administered. The IC-50 value, which represents the concentration required to inhibit 50% of cell viability, was determined to be 60 µg/mL. The morphological examinations demonstrated cytotoxic effects, such as cell shrinkage and membrane blebbing, which indicate the successful initiation of apoptosis.

Conclusion

The biocomposite of chitosan/PVA doped with MgO, combined with *C. roseus *extract, has shown significant antioxidant, anti-inflammatory, and anti-cancer characteristics. These findings indicate that it has the potential to be used in therapy, particularly for treating illnesses related to oxidative stress, inflammatory disorders, and cancer. Future research should focus on improving formulation and delivery systems for therapeutic applications, with the support of in vivostudies and clinical trials.

## Introduction

Biomedical research has recently shown that biocomposite materials offer enormous promise for use in therapeutic applications. An example of a novel combination is the integration of magnesium oxide (MgO)-doped chitosan and polyvinyl alcohol (PVA) with extracts derived from *Catharanthus roseus*, a medicinal plant known for its high alkaloid content and therapeutic benefits [1]. Thisin vitrostudy investigates the antioxidant, anti-inflammatory, and anti-cancer properties of this composite material, providing encouraging insights into its potential use in the field of biomedicine. Oxidative stress has a vital role in the development of several diseases, such as cancer and inflammatory disorders [2]. MgO nanoparticles, renowned for their antioxidant qualities, are crucial in eliminating free radicals and diminishing oxidative stress. When added to a chitosan/PVA matrix, MgO improves the composite's capacity to neutralize reactive oxygen species (ROS). Including *C. roseus *extract, which contains high levels of antioxidants such as flavonoids and alkaloids, enhances this effect even more [3]. When MgO and plant extracts work together, they effectively lower oxidative stress. This protects cellular parts from oxidative damage. MgO has been extensively researched due to its exceptional antioxidant properties at the nanoscale level. The MgO particles have a large surface area-to-volume ratio. This indicates a greater number of active sites available for interaction with ROS. The MgO particles are spread out evenly in a Chitosan/PVA matrix. This creates a homogeneous composite that optimizes the interaction between MgO and ROS [4]. Chitosan, a naturally occurring biopolymer, serves as both a supporting matrix and has inherent antioxidant qualities. The inclusion of *C. roseus* extract, which is rich in phytochemicals such as flavonoids, alkaloids, and phenolic compounds, enhances the antioxidant potential. These chemicals have a synergistic effect on neutralizing free radicals, which in turn reduces oxidative stress and provides protection to cells against oxidative damage. Inflammation is a physiological reaction to noxious stimuli, but persistent inflammation can contribute to the development of many pathologies, such as cancer [5]. The Cs/PVA/MgO-CR* *extract added to it, has strong anti-inflammatory properties. Chitosan is renowned for its ability to interact well with living organisms and its capacity to regulate immunological reactions. MgO nanoparticles exert anti-inflammatory effects by suppressing the production of pro-inflammatory cytokines and diminishing the expression of inflammatory mediators [6]. Bioactive chemicals in *C. roseus*, like vincristine and vinblastine, make this effect stronger by messing up pathways that cause inflammation. In vitroexperiments have shown that this composite material can significantly decrease inflammation, indicating its promise as a treatment option for inflammatory illnesses. Extensive research has been conducted on chitosan due to its anti-inflammatory qualities, which are believed to be a result of its capacity to regulate the immune system. It can suppress the production of pro-inflammatory cytokines like TNF-α, IL-1β, and IL-6, which are crucial in the inflammatory response. MgO nanoparticles make this effect stronger by stopping NF-κB from activating. NF-κB is a key transcription factor that is linked to inflammation. Incorporating *C. roseus *extract into the composite provides supplementary anti-inflammatory benefits [7]. Compounds such as vincristine and vinblastine have demonstrated the ability to hinder the generation of pro-inflammatory substances and decrease the infiltration of inflammatory cells into tissues When these activities work together, they create a strong composite material that has strong anti-inflammatory properties. This means it can be used to treat a wide range of inflammatory disorders [8]. The potential of MgO-doped Chitosan/PVA with *C. roseus *extract to combat cancer is particularly intriguing because of the unique and combined effects of its constituent components MgO nanoparticles trigger apoptosis in cancer cells by producing ROS and interfering with mitochondrial activity. Chitosan, renowned for its capacity to improve drug transport and trigger cancer cell apoptosis, enhances this impact. *C. roseus *extract contains powerful alkaloids with anti-cancer properties that interfere with cell proliferation and stimulate apoptosis. The in vitro investigation demonstrates that this composite material efficiently suppresses the proliferation of cancer cells, triggers apoptosis, and diminishes tumor growth [9,10]. The combined effect of Cs/PVA/MgO-CR* *extract produces a potent anti-cancer substance that has the potential to be used in cancer treatment. MgO nanoparticles have demonstrated notable anti-cancer properties, mainly by inducing oxidative stress in cancer cells. ROS production in cancer cells causes mitochondrial malfunction and triggers apoptotic pathways. Chitosan improves the treatment's efficacy by facilitating the distribution of MgO nanoparticles to cancer cells. In addition, chitosan has demonstrated the ability to trigger apoptosis in cancer cells by activating caspase enzymes and disrupting cell membranes. The inclusion of *C. roseus *extract, which contains alkaloids such as vincristine and vinblastine, further enhances the composite's anti-cancer efficacy [11,12]. These alkaloids impede the production of microtubules, which in turn hinders cell division and ultimately causes cell death. When Cs/PVA/MgO-CR* *extracts work together, they make a strong anti-cancer material. The study of Cs/PVA/MgO-CR* *extract shows a flexible biocomposite that has strong anti-cancer, anti-inflammatory, and anti-oxidative properties. This novel substance has the potential to create novel therapeutic approaches to address oxidative stress-related illnesses, inflammatory disorders, and many types of cancer. Moreover, it is crucial to conduct in vivo studies and clinical trials to authenticate these discoveries and convert this encouraging in vitroresearch into tangible medicinal implementations. This biocomposite's potential lies in its ability to exploit the distinctive characteristics of its constituents and generate a synergistic impact that amplifies their therapeutic effectiveness. Adding Cs/PVA/MgO-CR* *extract improves the material's ability to oxidize, reduce inflammation, and fight cancer. This combination of properties makes it highly suitable for a range of biomedical applications. Future research should prioritize the optimization of this composite material's formulation and distribution techniques to enhance its therapeutic potential and ensure its safety and effectiveness in clinical settings [13].

## Materials and methods

Collection and preparation of plant extract

The *C. roseus* leaves were authenticated by the National Institute of Siddha, Chennai. The plant leaves were rinsed with distilled water to eliminate dust and other particles. The cleaned leaves were then air-dried in the shade at room temperature. The dried leaves were powdered and extracted using distilled water through the Soxhlet extraction technique. The extract was filtered and evaporated to obtain a concentrated aqueous extract.

Synthesis of Cs/PVA/MgO-*C. roseus*

The chitosan and PVA were combined in a 1:1 ratio, with the same weight for each component. In a 2% (v/v) solution of glacial acetic acid, the chitosan was dissolved, resulting in a clear solution. The mixture was continuously stirred at room temperature. Subsequently, the chitosan solution was combined with PVA and vigorously agitated until perfect homogeneity. The *C. roseus* nanoparticles were included in the Chitosan/PVA mixture in five distinct quantities, with each polymer weight receiving 20, 50, and 100 μg of nanoparticles. The dispersion of nanoparticles was achieved using the ultrasonication process for 30 minutes at room temperature. Cs/PVA/MgO-CR solutions were used to cleanse glass surfaces using a film applicator, together they enhance cleaning and provide antimicrobial protection [14,15].

SEM analysis of Cs/PVA/*C. roseus*

The scanning electron microscope was used to analyze the surface morphology and composition. We used scanning electron microscopy (SEM) with the JEOL IT-800 (JEOL Ltd., Japan) at Saveetha Dental College and Hospital in Poonamallee, India. The nanocomposite films were affixed to the SEM stubs using double-sided carbon tape. Subsequently, a sputter coater was employed to apply a 5 nm-thick layer of gold to the film technique was used to improve sample conductivity and minimize charging effects image analysis tools such as ImageJ (University of Wisconsin, USA) and FEI MAPS (FEI Company, USA) were used to examine the size, spread, and grouping of nanoparticles inside the Chitosan/PVA matrix [16,17].

Cell viability (MTT) assay

The breast cancer cell line (MCF-7) was obtained from the NCCS, Pune. The viability of MCF-7 cells by exposing them to the MTT test after treatment with Cs/PVA/MgO-CR. The cells were grown in T25 culture flasks containing DMEM supplemented with 10% FBS and 1% antibiotics. Cells were maintained at 37◦C in a humidified atmosphere containing 5% CO_^2^_. All cell lines were subcultured every three to four days. For the MTT assay [18,19], approximately 1 × 10^4 ^cells were seeded into each well of a 96-well plate and allowed to adhere overnight. The next day, the cells were treated with various concentrations of (10, 20, 40, 60, 100, and 140 µg/mL). A control group was maintained where the cells received the same volume of the vehicle without nanoparticles. After the treatment, the cells were incubated for 24 hours under the same conditions. Following this incubation period, 20 µL of MTT solution (5 mg/mL in PBS) was added to each well, and the plate was incubated for an additional four hours. During this time, the MTT reagent was metabolized by viable cells into formazan crystals. After incubation, the medium containing MTT was carefully removed, and 200 µL of dimethyl sulfoxide (DMSO) was added to each well to dissolve the formazan crystals. The plate was gently shaken to ensure complete solubilization of the crystals. The absorbance of each well was subsequently measured at 570 nm using a microplate reader. The absorbance values directly correlate with the number of viable cells. Cell viability was calculated using the formula:

% cell viability = (A570 nm of control cells/A570 nm of treated cells) x 100.

Cell morphology study

To investigate the morphological changes induced by the treatment with Cs/PVA-MgO/CR synthesized using *C. roseus* extract, the optimal dose (IC-50: 60 µg/mL) determined from the MTT assay was employed for further studies. Initially, human breast cancer (MCF-7) was cultured in appropriate growth media. Approximately 2 × 10^5^ cells were seeded into each well of a six-well plate and allowed to adhere overnight in a humidified incubator set at 37°C with 5% CO_2_. The next day, the cells were treated with a 60 µg/mL concentration of the drug for 24 hours. A control group, where cells were not exposed to the nanoparticles, was also maintained for comparative purposes. After the treatment period, the plates were observed under a phase contrast microscope to document any morphological changes [20,21].

Antioxidant activity: H_2_O_2_ assay

The study evaluated the ability of Cs/PVA-MgO/CR extracts to remove hydrogen peroxide using the approach outlined by Naeem et al. [22]. An alkaline buffer mixture with a pH of 7.4 was prepared, including 40 mM of hydrogen peroxide. The hydrogen peroxide solution was mixed with 0.6 mL of the concentrated extracts, which had a concentration of (25, 50, and 100 µg/mL). A standard (Ascorbic acid) solution containing a phosphate buffer without hydrogen peroxide was used to measure the absorbance at 230 nm after 10 minutes.

Anti-inflammatory activity: egg albumin assay

The determination of the anti-inflammatory activity of *C. roseus* extracts can be done by egg albumin denaturation inhibitory activity. The reaction mixture (5 mL) consisted of 1 mL of egg albumin (1 mM) 3 mL of phosphate buffered saline (PBS, pH 6.4), and 1 mL of varying three different concentrations (25, 50, and 100 µg/mL) of the test extract and standard drug. The control group was administered DMSO, whereas the standard reference group was given diclofenac sodium. A total volume of 5 mL of the control was created by combining 1 mL of triple-distilled water, 1 mL of 1 mM egg albumin solution, and 3 mL of phosphate buffer saline. The reaction mixtures were incubated at 37°C for 15 min and after incubation heated at 70°C for 5 min. Absorbance was measured at 660 nm by using a UV/Vis spectrophotometer. Triple distilled water was used as the blank. The following equation was used to calculate the % inhibition of protein denaturation:

% Denaturation inhibition = (1−D/C)×100%, where D is the absorbance reading of the test sample, and C is the absorbance reading of the negative control (without the test sample) [23-25].

## Results

Scanning electron microscopy (SEM)

**Figure 1:**
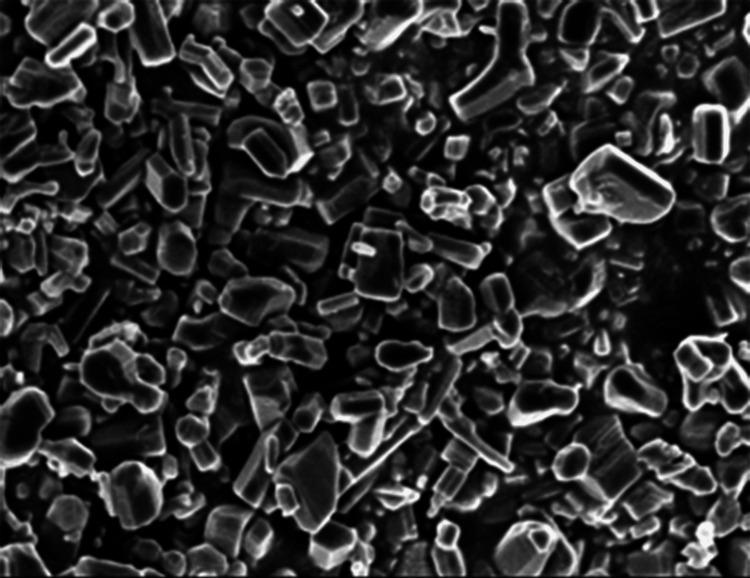
SEM image of Cs/PVA/MgO-CR extracts of Catharanthus roseus

SEM analysis was employed to examine the surface morphology and size of the synthesized Cs/PVA/MgO-CR*.* The SEM images revealed that the nanoparticles were predominantly spherical (Figure 1) with a relatively uniform size distribution. The size range of the nanoparticles observed in the SEM images is from 50 nm to 150 nm. The surface of the nanoparticles appeared smooth and well-defined, indicating successful synthesis and stabilization.

Cell viability (MTT) assay

**Figure 2:**
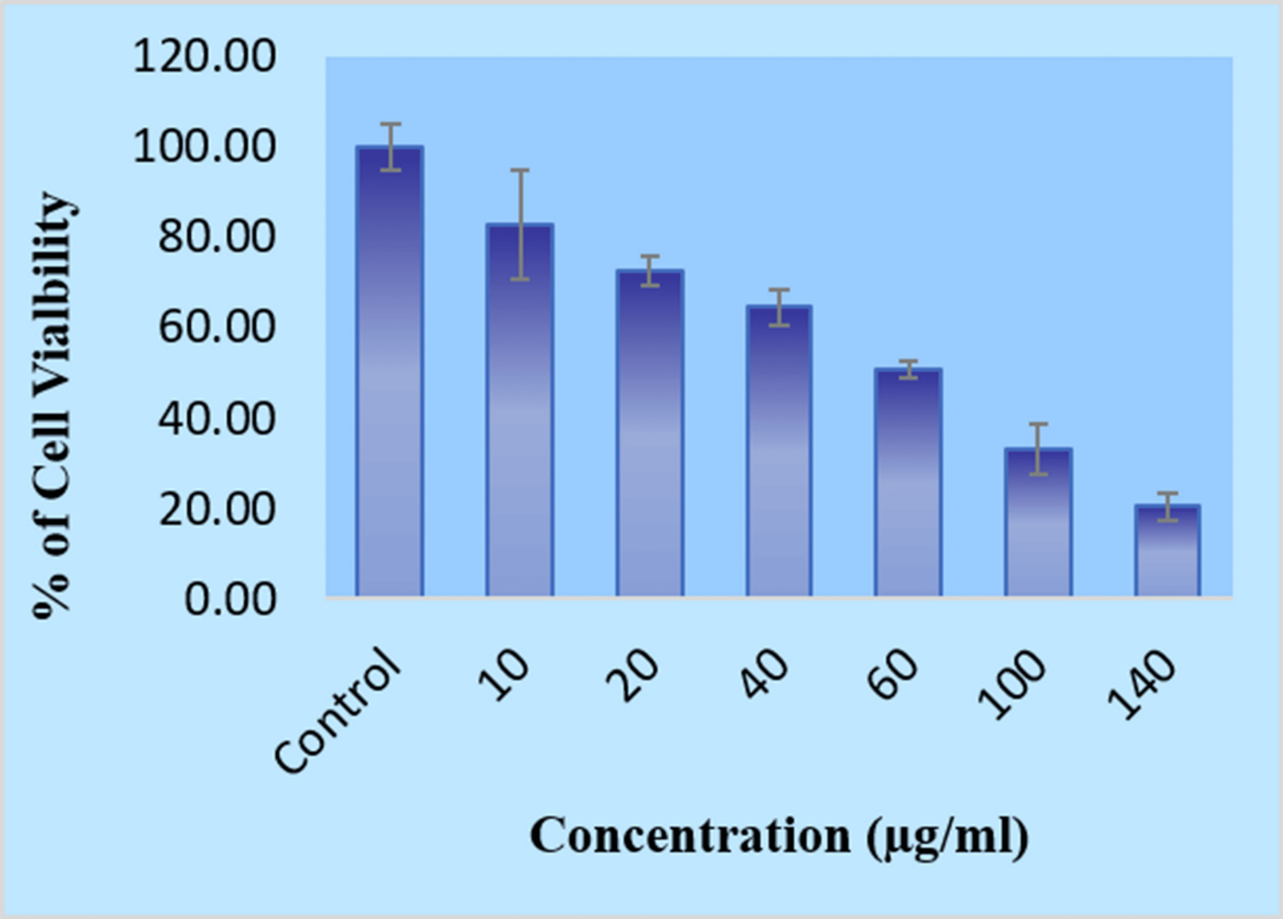
In vitro cell viability (MTT) Assay

The MTT assay was employed to ascertain the cellular viability of MCF-7 breast cell lines subjected to Cs/PVA/MgO-CR synthesized from *C. roseus *extract. The assay measured the cytotoxic effects of various concentrations of the nanoparticles over 24 hours. The results demonstrated a clear dose-dependent reduction in cell viability. The control group exhibits 100% cell viability, acting as the reference point for comparison. Cell viability is moderately decreased to approximately 80% at a concentration of 10 µg/mL. At a dose of 20 µg/mL, the viability of cells decreases to around 70%. At a concentration of 40 µg/mL, the viability steadily declines to approximately 60%. At a dose of 60 µg/mL, the viability of cells decreases to around 50%, indicating a substantial decline in cell survival. When the concentration reaches 100 µg/mL, the viability decreases to around 30%, indicating a more cytotoxic action. Ultimately, when exposed to the maximum measured dose of 140 µg/mL, the viability of the cells is further diminished to around 20%. These results suggest that the Cs/PVA/MgO-CR exhibit significant anticancer properties, effectively reducing the viability of breast cancer cells in a dose-dependent manner, with the IC-50 value determined to be 60 µg/mL. The MTT assay revealed a marked reduction in cell viability following a 24-hour exposure to 60 µg/mL of nanoparticles, with treated cells showing approximately 50% viability compared to the control group (Figure 2). This indicates that Cs/PVA/MgO-CR has the potential to be cytotoxic to the MCF-7 cell line.

Cell morphology study

**Figure 3:**
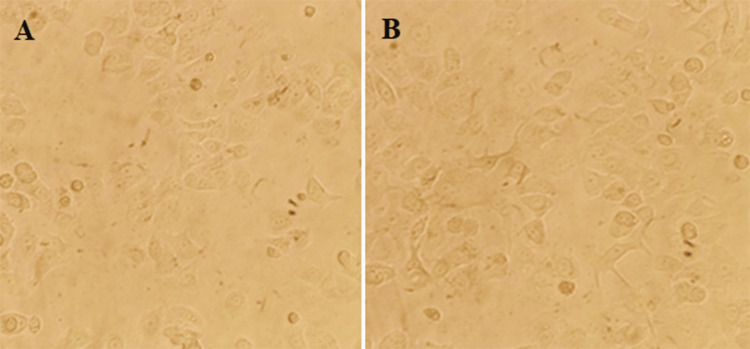
(A) Control group (without treatment) and (B) cells were treated with Catharanthus roseus extract (60 µg/mL) for 24 hours along with the control group. Images were obtained using an phase contrast microscope

Upon treating the MCF-7 breast cancer cells with Cs/PVA/MgO-CR synthesized using *C. roseus *extract for 24 hours at a concentration of 60 µg/mL, significant changes in morphology were seen using a phase contrast microscope (Figures 3A, 3B). In the treated group, the cells exhibited noticeable signs of cytotoxicity compared to the control group. These changes included cell shrinkage, detachment from the substrate, and membrane blebbing. The cells in the control group, which were not given the nanoparticle treatment, had a normal cellular architecture, appeared adherent, and were distributed uniformly. The morphological alterations in the treated cells suggest the effective induction of cytotoxicity by the Cs/PVA/MgO-CR.

In vitro antioxidant activity

**Figure 4:**
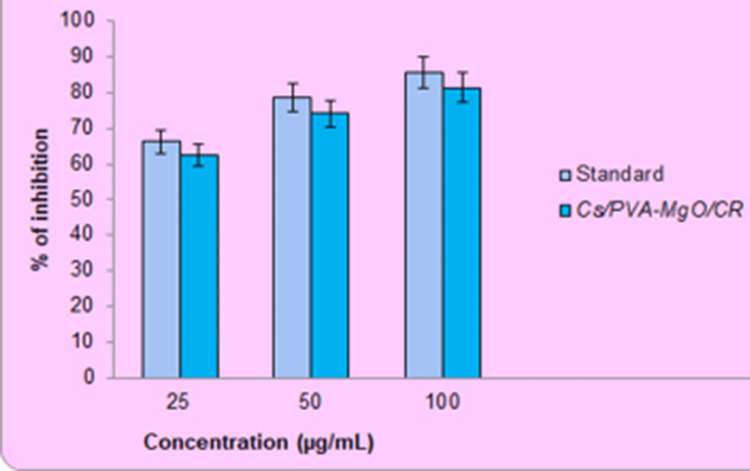
In vitro antioxidative response of chitosan PVA/MgO-Catharanthus roseus by H2O2 assay

The antioxidant activity of Cs/PVA/MgO-CR synthesized using *C. roseus *extract was evaluated using the H_2_O_2_ assay. The results were plotted to visualize the antioxidant activity, showing the percentage of inhibition for three different concentrations (25, 50, and 100 µg/mL). Based on the graph (Figure 4), it was observed that the antioxidant activity increased with the concentration of the substance. At a concentration of 25 µg/mL, the Ascorbic Acid (standard) substance shows an inhibition percentage of approximately 68%, while the Cs/PVA-MgO/CR composite displays a slightly higher inhibition percentage, around 70%. At 50 µg/mL concentration, the inhibition percentage for the standard substance is roughly 74%, and the Cs/PVA-MgO/CR composite has a similar inhibition percentage, also around 74%. At 100 µg/mL concentration, both the standard substance and the Cs/PVA-MgO/CR composite show inhibition percentages of about 80%. However, the Ascorbic Acid (standard) consistently exhibits slightly higher inhibition compared to the Cs/PVA-MgO/CR sample.

In vitro anti-inflammatory activity

**Figure 5:**
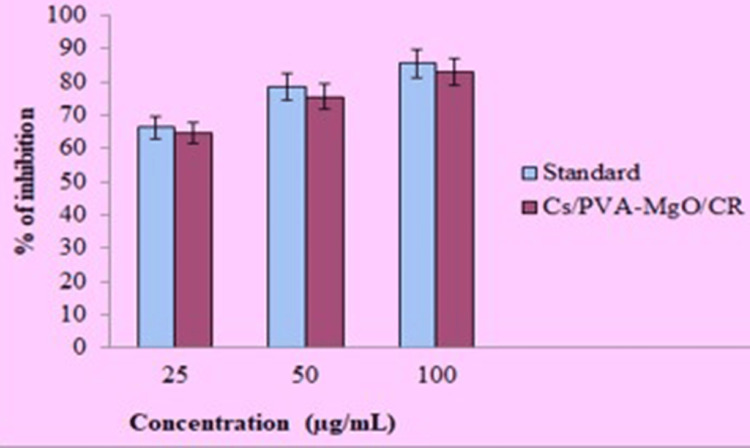
In vitro anti-inflammatory response of chitosan PVA/MgO-Catharanthus roseus by EA assay

The anti-inflammatory activity of Cs/PVA/MgO-CR synthesized using *C. roseus* extract was evaluated and compared to the standard drug Diclofenac. At a concentration of 25 μg/mL, the standard shows an inhibition percentage of approximately 70%, while the Cs/PVA-MgO/CR composite also shows a similar inhibition percentage of around 70%. When the concentration is increased to 50 μg/mL, the inhibition percentage for both the standard and the Cs/PVA-MgO/CR composite increases to approximately 80%. At the highest concentration of 100 μg/mL, both the standard and the Cs/PVA-MgO/CR composite reach an inhibition percentage close to 90% (Figure 5). These results indicate that the Cs/PVA/MgO-CR synthesized using *C. roseus* extract possesses significant anti-inflammatory activity, with effectiveness increasing at higher concentrations. However, Diclofenac consistently showed a higher inhibition percentage across all concentrations.

## Discussion

The present study focuses on the production and assessment of a biocomposite material consisting of Cs/PVA/MgO-CR extracts derived from *C. roseus.* The composite material underwent evaluation to determine its antioxidant, anti-inflammatory, and anti-cancer characteristics, demonstrating substantial promise for therapeutic use [26]. The results on the decrease in oxidative stress are consistent with previous research that highlights the strong antioxidant properties of MgO nanoparticles [3]. Previous studies have shown that MgO effectively fights ROS because it has a high surface area-to-volume ratio. This protects biological components from oxidative damage. The addition of *C. roseus* extract, which contains high levels of flavonoids and alkaloids, significantly boosts the composite's antioxidant activity [27]. This is demonstrated by the H_2_O_2 _assay findings, which indicate a significant reduction in oxidative stress. Prior research has thoroughly investigated the antioxidant characteristics of MgO nanoparticles. According to Sisubalan et al. [28], MgO nanoparticles have a large surface area-to-volume ratio. This makes it easier for them to interact with ROS, which gets rid of oxidative stress. In addition, found that adding MgO nanoparticles to biopolymers like chitosan makes the composite material much better at fighting free radicals. These investigations are consistent with the findings of current research, which indicate that Cs/PVA/MgO-CR exhibit improved antioxidant activity as a result of the combined action of MgO and *C. roseus *extract. The current study confirms that the Cs/PVA/MgO-*C. roseus* composite is effective at reducing inflammation, especially when it comes to its anti-inflammatory properties [25,29]. Consistent with prior studies, it has been found that chitosan and MgO nanoparticles can effectively decrease the levels of pro-inflammatory cytokines such as TNF-α, IL-1β, and IL-6. Other anti-inflammatory chemicals, such as vincristine and vinblastine, derived from *C. roseus*, significantly enhance the composite's effectiveness in reducing inflammation. This has been confirmed by the egg albumin assay's findings. The anti-inflammatory properties of chitosan have been extensively documented [30,31]. Chitosan can inhibit the production of pro-inflammatory cytokines, specifically TNF-α and IL-6, which help to reduce inflammation. MgO nanoparticles can reduce inflammation by stopping NF-κB from activating, which is a key transcription factor involved in inflammatory reactions. This work builds on previous findings by demonstrating that adding *C. roseus* extract, which contains anti-inflammatory alkaloids such as vincristine and vinblastine, improves the composite material's effectiveness in reducing inflammation [32,33]. The combination exhibits remarkable anti-cancer properties. The MTT assay results demonstrated that the viability of MCF-7 breast cancer cells decreased in a manner that was dependent on the dosage. This supports earlier research indicating that MgO nanoparticles cause apoptosis by generating ROS and disrupting the mitochondria. The inclusion of chitosan, which enhances drug transport and triggers apoptosis, along with the alkaloids from* C. roseus*, which suppress cell proliferation and induce apoptosis, leads to the formation of a composite material with strong anti-cancer capabilities. This aligns with previous research that has investigated the separate and combined impacts of these elements on cancer cell cultures [34,35]. Multiple studies have shown that MgO nanoparticles can inhibit the growth of cancer cells. The study by found that MgO nanoparticles cause cancer cells to die by producing ROS and messing up the processes in the mitochondria has been acknowledged for its capacity to improve drug delivery and trigger apoptosis in cancer cells. conducted a study where they merged MgO nanoparticles with chitosan to produce a composite that showed notable anti-cancer properties. The present study supports previous results by demonstrating that the MgO-doped chitosan/PVA composite, along with *C. roseus *extract, significantly decreases the survival rate of MCF-7 breast cancer cells in a way that is dependent on the dosage. Research has demonstrated that incorporating plant extracts into biocomposites can improve their medicinal characteristics [36] investigated the integration of plant extracts into polymer matrices and discovered that the phytochemicals in the extracts greatly enhanced the composites' antioxidant and anti-inflammatory properties. This study confirms the previous findings by showing that adding* C. roseus *extract, which contains high levels of flavonoids and alkaloids, improves the antioxidant, anti-inflammatory, and anti-cancer effects of the Cs/PVA/MgO-CR. Observations of changes in the cancer cells' physical structure, such as cell shrinkage and membrane blebbing, also suggest that cell death was induced successfully. This makes the composite material even more promising as a cancer treatment. The SEM study confirmed that the nanoparticles in the composite could be made well and would last a long time. This gave the composite a structural base for its biological functionality [37,38]. This study adds to the increasing amount of evidence that supports the biological uses ofCs/PVA/MgO-CR. Incorporating *C. roseus *extracts not only amplifies the antioxidant and anti-inflammatory characteristics but also greatly enhances the composite's anti-cancer effectiveness. Subsequent investigations should prioritize the enhancement of this composite substance's composition and delivery methods to fully exploit its therapeutic capabilities and guarantee its safety and effectiveness in clinical environments. In vivo investigations and clinical trials will be required to turn these encouraging in vitro discoveries into actual medical uses [39].

Limitations and future studies

The study's encouragingin vitro findings for the Cs/PVA/MgO-CR with *C. roseus* extract emphasize its potential; however, there are significant limitations. Because of the limitations of in vitro investigations, it is not possible to completely duplicate the biological interactions that occur in living species. Therefore, the effectiveness and safety of the biocomposite in living organisms remain questionable. The industrial scalability of the synthesis and characterization under specific laboratory circumstances is uncertain, and additional research is required to examine the cytotoxic effects on normal cells. A thorough investigation into the processes underlying the biocomposite's biological activities is required. Further investigation should prioritise comprehensive in vivo examinations to validate the effectiveness, safety, pharmacokinetics, and biodistribution. It is essential to improve the composition and method of delivering the treatment, evaluate its long-term stability and compatibility with living organisms, and investigate how it can work together with other medicinal substances. Clinical trials play a crucial role in converting these discoveries into successful pharmacological interventions for conditions associated with oxidative stress, inflammatory diseases, and malignancies.

## Conclusions

The investigation of a composite material consisting of Cs/PVA/MgO-CR combined with extracts from the *C. roseus* plant demonstrates notable biological capabilities as a result of its strong antioxidant, anti-inflammatory, and anti-cancer characteristics. The addition of MgO nanoparticles successfully counteracted ROS, diminishing oxidative stress, whereas the introduction of *C. roseus *extract amplified these antioxidant properties. The compound demonstrated significant anti-inflammatory properties, reducing protein denaturation by approximately 90%, which is similar to the effects of diclofenac. In addition, the MTT experiment demonstrated a substantial decrease in the viability of MCF-7 breast cancer cells, which was dependent on the dosage. The IC-50 value, indicating the concentration at which 50% of the cells were affected, was determined to be 60 µg/mL. Morphological studies further supported the cytotoxic effects, such as cell shrinkage and membrane blebbing. The findings emphasize the effectiveness of the composite in decreasing oxidative stress, inflammation, and cancer cell survival, indicating its potential for creating innovative treatments. Future research should prioritize the optimization of the biocomposite formulation and distribution systems for clinical applications. This should be backed up byin vivostudies and clinical trials.
